# Inflammatory Cytokines and Radiotherapy in Pancreatic Ductal Adenocarcinoma

**DOI:** 10.3390/biomedicines10123215

**Published:** 2022-12-12

**Authors:** Sylvia S. W. Ng, Laura A. Dawson

**Affiliations:** 1 Department of Radiation Oncology, Odette Cancer Centre, Sunnybrook Health Sciences Centre, Toronto, ON M4N 3M5, Canada; 2 Department of Radiation Oncology, Temerty Faculty of Medicine, University of Toronto, Toronto, ON M5T 1P5, Canada; 3 Radiation Medicine Program, Princess Margaret Cancer Centre, University Health Network, 610 University Avenue, Toronto, ON M5G 2C1, Canada

**Keywords:** pancreatic cancer, stereotactic ablative radiotherapy, interleukins, inflammatory cytokines, tumor–stromal interactions

## Abstract

Pancreatic ductal adenocarcinoma (PDAC) remains a therapeutic challenge in clinical oncology. Surgery is the only potentially curative treatment. However, the majority of PDAC patients present with locally advanced/unresectable or metastatic disease, where palliative multiagent chemotherapy is the first-line treatment with the therapeutic intent to delay progression and prolong survival. For locally advanced/unresectable pancreatic cancer patients who are treated with chemotherapy, consolidative radiotherapy in the form concurrent chemoradiation or stereotactic ablative radiotherapy improves locoregional control and pain/symptom control. To improve clinical outcomes of PDAC patients, there is a dire need for discoveries that will shed more light on the pathophysiology of the disease and lead to the development of more efficacious treatment strategies. Inflammatory cytokines are known to play a role in mediating tumor progression, chemoresistance, and radioresistance in PDAC. A PubMed search on published articles related to radiotherapy, inflammatory cytokines, and pancreatic cancer patients in the English language was performed. This article primarily focuses on reviewing the clinical literature that examines the association of inflammatory cytokines with clinical outcomes and the effects of radiotherapy on inflammatory cytokines in PDAC patients.

## 1. Introduction

Over 90% of malignant pancreatic tumors are adenocarcinomas derived from the ductal epithelium of the exocrine pancreas [1]. Pancreatic ductal adenocarcinoma (PDAC) is the second most common gastrointestinal malignancy and the fourth leading cause of cancer death in the United States and Canada [2,3]. While improvements in overall survival and quality of life have been demonstrated in patients with other solid tumor malignancies such as breast, colorectal, and lung cancers over the past decade secondary to the use of targeted therapy and immunotherapy, patients with PDAC continue to have a poor prognosis with a five-year survival rate of 11% for all stages combined, 42% for those with localized disease, 14% for those with regional disease, and 3% for those with metastatic disease [2].

## 2. Current Treatment Modalities for Pancreatic Cancer 

Surgical resection with negative margins (R0 resection) is the only potentially curative treatment. However, only 15–20% of PDAC patients present with resectable disease and disease recurrence following a Whipple resection is common [4,5,6,7]. The majority of PDAC patients present with locally advanced/unresectable or metastatic disease [8], which is not curable. Furthermore, most PDAC are diagnosed in patients at the age of 70 or above [3], who have significant co-morbidities that render them medically inoperable. Treatment options for these patients include chemotherapy and radiotherapy. Multiagent chemotherapy using FOLFIRINOX (folinic acid, 5-fluorouracil, irinotecan, oxaliplatin) or gemcitabine/nab-paclitaxel has significantly improved overall survival by 2 to 4 months compared to single-agent gemcitabine in metastatic PDAC [9,10], and is the current standard-of-care treatments. By extrapolation of the data, the 2022 National Comprehensive Cancer Network (NCCN) guidelines [11] recommended the use of FOLFIRINOX and gemcitabine/nab-paclitaxel in locally advanced/unresectable PDAC.

### Conventional Radiotherapy and Stereotactic Ablative Radiotherapy

It should be noted that approximately 30% of PDAC patients die with locally destructive disease, while 70% die with widespread metastatic disease [12]. Locoregional disease progression often causes substantial pain secondary to celiac plexus infiltration and complications such as gastric outlet obstruction or duodenal bleeding, all of which diminish the quality of life of PDAC patients. As systemic therapy becomes more efficacious in controlling distant metastasis, providing durable locoregional control with radiotherapy is anticipated to become increasingly important in the long-term management of PDAC. 

Conventional radiotherapy (50.4–54 Gy in 1.8–2 Gy per fraction) given concurrently with capecitabine or stereotactic ablative radiotherapy alone (SABR; 33–40 Gy in 6.6–8 Gy fractions) is used in the management of locally advanced/unresectable PDAC with the intent to maximize locoregional control. The role of concurrent chemoradiation in the management of locally advanced/unresectable PDAC is a matter of debate due to conflicting results from early studies with respect to whether or not adding concurrent chemoradiation to chemotherapy confers survival benefits compared to chemotherapy alone [13,14,15,16]. In the LAP07 phase III randomized trial [17] where locally advanced PDAC patients were treated with induction gemcitabine +/− erlotinib followed by concurrent chemoradiation using conventional fractionation (54 Gy in 30 fractions + capecitabine 800 mg/m^2^ twice daily on days of radiation) or gemcitabine +/− erlotinib alone, significant decrease in local recurrence was reported in the former compared to the latter (32% vs. 46%, *p* = 0.03), while no difference in overall survival was observed between the two arms (15.2 vs. 16.5 months, *p* = 0.83). 

Improved locoregional control can be achieved by escalating radiation dose using modern treatment techniques such as SABR and magnetic resonance imaging (MRI)-guided adaptive radiotherapy [18]. Indeed, patients with locally advanced and locally recurrent PDAC who were treated with SABR had excellent local tumor control with acceptable toxicity [19,20,21,22,23,24,25]. In addition to providing local control, SABR alleviates cancer-related pain on the EORTC QLQ-PAN26 questionnaire and improves quality of life on the QLQ-C30 global QoL scores at 4 months post treatment in patients with borderline resectable and locally advanced PDAC [19,22]. The 2022 NCCN guidelines included both concurrent chemoradiation and SABR as radiotherapy options for locally advanced/unresectable PDAC. In the 2019 American Society for Radiation Oncology (ASTRO) clinical practice guidelines for radiation therapy in pancreatic cancer [26], a definitive treatment regimen consisting of systemic chemotherapy followed by concurrent chemoradiation using conventional fractionation or dose-escalated radiotherapy, or multi-fraction SABR alone, was conditionally recommended in locally advanced disease that is not appropriate for downstaging to eventual surgery. 

Radiation dose escalation in PDAC treatment is limited by the close anatomic proximity of the pancreatic tumor to the dose-limiting, radiosensitive luminal gastrointestinal structures, namely the duodenum, small bowel, stomach, and large bowel. Image-guided radiotherapy in the modern era most commonly uses cone beam computed tomography (CBCT) for daily image guidance and radiation treatment verification. CBCT has poor soft tissue contrast and is suboptimal for image-guided SABR in the treatment of PDAC because the pancreatic tumor cannot be clearly distinguished from the surrounding normal luminal gastrointestinal structures. The MR-LINAC (MRL), which combines MRI with a linear accelerator (LINAC), provides superior soft tissue contrast compared to CBCT [27] and in turn, enables more accurate delineation of soft tissue target(s) and normal luminal gastrointestinal organs. This capability makes the MRL highly suited for safe dose escalation of SABR in the treatment of PDAC. More importantly, using the MRL, the radiation treatment plan can be adapted in real-time on the day of treatment based on the patient’s luminal gastrointestinal anatomy of the day just prior to radiation delivery. Dose delivery to the intended target(s) is thus maximized for cancer control while minimizing toxicities to the surrounding luminal gastrointestinal organs. Chuong et al. [28] demonstrated 1-year local control, progression-free survival, and overall survival of 87.8%, 52.4%, and 58.9%, respectively, in patients with inoperable PDAC who received induction chemotherapy followed by SABR to a total dose of 50 Gy in five fractions (biological equivalent dose or BED = 100 Gy) delivered using MRI-guided adaptive radiation therapy; updated results by the same authors recently reported median progression-free survival of 20 months and median overall survival of 23 months; and 2-year local control, progression-free survival, and overall survival of 68.8%, 40%, and 45.5%, respectively, in this cohort of PDAC patients [29]. Acute and late grade 3 or higher toxicity rates were both 4.8% [29]. Furthermore, in locally advanced PDAC patients who received induction chemotherapy followed by ablative, escalated-dose radiotherapy to a total dose of 75 Gy in 25 fractions (BED = 97.5 Gy) or 67.5 Gy in 15 fractions (BED = 97.88 Gy), median overall survival from diagnosis and radiotherapy were reported to be 26.8 months and 18.4 months, respectively; 1-year and 2-year overall survival from radiotherapy were 74% and 38%, respectively; 1-year and 2-year cumulative incidence of locoregional failure were 17.6% and 32.8%, respectively; grade 3 upper gastrointestinal bleeding occurred in 8% of treated patients with no grade 4 to 5 toxicity [30]. In addition, a single 25-Gy fraction targeting the celiac plexus has been shown to improve pancreatic cancer-associated celiac pain and decrease the use of opioid analgesic [31]. Taken together, ablative doses of radiotherapy (BED = 100 Gy) provide improved tumor control and survival compared to conventional radiotherapy in PDAC patients. These data are very encouraging, and a Phase III randomized controlled trial titled “locally advanced pancreatic cancer treated with ablative stereotactic MRI-guided adaptive radiation therapy (LAP-ABLATE)” has been launched recently with the study design to demonstrate superior overall survival in PDAC patients who receive chemotherapy followed by dose-escalated, MRL-based SABR compared to those who receive chemotherapy alone. 

While technological advances in ablative radiotherapy delivery hold promise to improve the prognosis of PDAC, the biological mechanisms of action of ablative radiotherapy have not been fully elucidated. Elevation of specific cytokines has been shown to portend poorer survival in PDAC [32,33,34,35,36,37,38,39]. It is conceivable that understanding how ablative radiotherapy affects the pancreatic tumor microenvironment in situ via different cytokines may pave the way for the improvement of therapeutic efficacy of existing treatments and the development of new combinations of treatments. This article focuses on reviewing the association of inflammatory cytokines with clinical outcomes and the effects of radiotherapy on circulating inflammatory cytokines in PDAC patients. 

## 3. Inflammatory Cytokines and Clinical Outcomes

**Figure 1:**
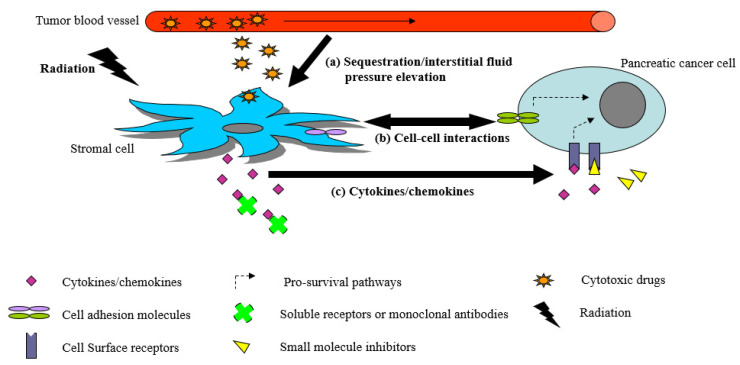
Potential mechanisms by which stromal cells such as pancreatic cancer-associated fibroblasts (CAFs) and immune cells contribute to radiotherapy/chemotherapy resistance in pancreatic cancer. (**a**) Given their large population in the pancreatic tumor stroma, CAFs may sequester cytotoxic drugs. CAFs often exhibit increased contractility and exert increased tension on the modified extracellular matrix (ECM) in the pancreatic tumor mass, resulting in elevated intratumoral interstitial pressure and decreased cytotoxic drug delivery to pancreatic cancer cells. (**b**) Physical interactions between stromal cells and pancreatic cancer cells may increase the activity of pro-survival pathways in the latter. (**c**) Stromal cells are avid producers of many growth factors, cytokines/chemokines, and ECM proteins. Binding to some of these molecules to their respective receptors on pancreatic cancer cells may also increase pro-survival signaling, leading to increased protection from the cytotoxic effects of radiotherapy/chemotherapy. Therefore, blocking the action of specific soluble factors and cytokines released by stromal cells with monoclonal antibodies or small molecule inhibitors represent viable strategies to enhance radiosensitivity/chemosensitivity of pancreatic cancer cells.

It is recognized that tumor progression results not only from accumulation of genetic mutations in cancer cells but also from dynamic, reciprocal communication between cancer cells and the stroma [40,41]. Cancer cells alter the stromal environment by producing growth factors such as transforming growth factor-β (TGF-β), platelet-derived growth factor (PDGF), basic fibroblast growth factor (bFGF), and vascular endothelial growth factor (VEGF), as well as proteases [42,43]. This modified “reactive” stroma or desmoplasia, consisting of activated fibroblasts (also known as cancer-associated fibroblasts, CAFs), vascular endothelial cells, pericytes, inflammatory and immune cells, in turn provides a myriad of soluble factors and cytokines to promote tumor growth, angiogenesis, invasion, and metastasis (Figure 1). Cytokines are small soluble proteins, peptides, or glycoproteins that are produced by various cells (e.g., immune, endothelial, and epithelial cells) in the body [44]. They are mediators of cellular signaling, and are produced in response to immune, inflammatory, or infectious stimuli. In addition, cytokines are involved in processes such as cell proliferation and death, as well as tumor initiation and progression [45]. They are vital in coordinating and modulating the body’s response to external and internal stimuli. Cytokines can be grouped according to their primary functions, or by the cells that produce them. Many cytokines are multi-functional and can be produced by different cell sources under different conditions [46]. There has been great interest in evaluating cytokines in different disease states as prognostic markers [47,48], biomarkers to monitor response and toxicities to anticancer treatments [48,49], and potential therapeutic targets [50,51]. 

### 3.1. Association of Various Inflammatory Cytokines with Prognosis 

Notably, PDAC is characterized by a strong desmoplastic reaction in association with extensive fibroblast proliferation and modified extracellular matrix deposition. In some cases, this fibrovascular stroma makes up greater than 90% of the total pancreatic tumor mass [52]. In addition, the tumor microenvironment of PDAC is generally immunosuppressive, consisting of regulatory tumor associated macrophages (TAMs) and regulatory T cells (Treg), [53,54,55]. Reciprocal signaling between pancreatic cancer cells, CAFs, TAMs, Treg, and other stromal cells are mediated in part by inflammatory cytokines (Figure 1), and have been demonstrated in a large volume of preclinical in vitro and in vivo studies using co-cultured cells, genetically engineered mice, organoids, and human pancreatic cancer xenografts implanted in immunodeficient mice [54,55,56,57]. Whether or not the mechanistic pathways of tumor–stromal crosstalk that were defined in cells/organoids and animal models reflect what is happening in the pancreatic tumor microenvironment of patients are unclear. Published clinical studies often report the association between high or low plasma levels of specific cytokines with clinical outcomes. In a large prospective study (n = 446) [33], PDAC patients with higher plasma levels of interleukin-6 (IL-6), macrophage inhibitory cytokine-1 (MIC-1), tumor necrosis factor-ɑ (TNF-ɑ), and C-reactive protein were shown to have significantly shorter median survival than those with lower levels of all of these four inflammatory markers (3.7 vs. 19.2 months, *p* < 0.0001). Increased circulating IL-6 levels were shown to correlate with shorter survival, poor performance status, cachexia, and weight loss in PDAC patients [32,33,34,35,36]. Elevated circulating IL-6, IL-8, IL-10, and TNF-ɑ levels were linked to shorter survival [37,38,39], while elevated plasma IL-11 levels were associated with longer survival in PDAC patients [58]. In another study [59], PDAC patients with high serum levels of IL-6 and IL-1𝛽 demonstrated inferior median overall survival (79 vs. 306 days, *p* < 0.001), progression-free survival (46 vs.158 days, *p* < 0.001), and tumor control rate following gemcitabine (20% vs. 76%, *p* < 0.001) than those with low levels of both cytokines. Interestingly, PDAC patients with lower circulating IL-1RA levels had worse survival than those with higher circulating IL-1RA levels [32]. High serum IL-1RA concentration after one cycle of FOLFIRINOX was found to be an independent predictor of radiographic tumor response to FOLFIRINOX in PDAC patients [60]. A phase IB study to assess the safety of canakinumab (anti-IL-1𝛽 antibody), spartalizumab (anti-PD-1 antibody), and gemcitabine/nab-paclitaxel in metastatic PDAC (PanCAN-SR1, NCT04581343), and a phase II study to evaluate the efficacy of Anakinra (IL-1RA) plus chemotherapy in the perioperative setting (NCT04926467) are underway. While the reasons as to why high or low levels of specific concentrations of cytokines in the circulation of PDAC patients portend the worst clinical outcomes remain to be fully understood, emerging data suggest that tumor response or resistance to chemotherapy or radiotherapy is in part modulated by the interplay between immunostimulatory, tumor-inhibiting cytokines and their immunosuppressive, tumor-promoting counterparts. 

### 3.2. Interleukin-6

Interleukin-6 (IL-6) is perhaps one of the most well studied inflammatory cytokines. It is understood that IL-6 signaling is mediated by a receptor system that consists of an 80 kD ligand-binding, membrane-bound IL-6 receptor (mIL-6R), and a 130 kD signal transducing chain called gp130. gp130 is also membrane-bound and is expressed ubiquitously in vivo [61]. mIL-6Rs are expressed in hepatocytes, pancreatic alpha cells, neutrophils, monocytes, macrophages, as well as B and T cells [62,63]. Binding of IL-6 to mIL-6R recruits two gp130 molecules whose activation is followed by phosphorylation of Janus kinases (Jak1, Jak2, Tyk2) and recruitment of the signal transducers and activators of transcription STAT1 and STAT3 [64]. Phosphorylation of STATs then leads to their dimerization and nuclear translocation, thereby initiating the transcription of numerous genes such as VEGF [65], and the antiapoptotic proteins Bcl-2 and Bcl-X_L_ [66,67]. Depending on the cell type, IL-6 can also activate the MAPK and PI3K/Akt pathways to promote cell survival [68,69,70]. A soluble form of IL-6R (sIL-6R) also exists as a result of proteolytic cleavage of mIL-6R by the metalloproteinases ADAM10 and ADAM17 [71,72,73] or translation of alternatively spliced mRNA [74]. Unlike other soluble receptors which often act as antagonistic decoys to prevent ligands from binding to the corresponding membrane-bound receptors, sIL-6R is agonistic and transmits signals following IL-6 binding via a process called trans-signaling [64,75]. The IL-6/sIL-6R complex can bind to and activate gp130, and trigger downstream signaling effectors [76]. Therefore, cancer cells that do not express IL-6 or mIL-6R can still respond to IL-6 if IL-6 and sIL-6 are produced by stromal cells. It should be noted that the activity of the IL-6/sIL-6R complex is tightly regulated and antagonized by an endogenous, soluble form of gp130 (sgp130) [77]. Circulating sgp130 binds to the IL-6/sIL-6R complex and blocks signaling via the membrane-bound gp130 [78]. Considering that IL-6 and sIL-6R levels are often elevated in various pathological conditions including pancreatic cancer, endogenous sgp130 may not be sufficient to keep the overactive IL-6 trans-signaling in check. Indeed, upregulation of ADAM17 and downregulation of mIL-6R were observed in a colon cancer model [73] and in colon cancer patients [79], leading to increased cleavage of the mIL-6R and release of the sIL-6R from the tumor cells. Subsequently, the IL-6/sIL-6R complex activated membrane-bound gp130 to increase STAT3 phosphorylation in the tumor cells [73]. A recombinant fusion protein with the extracellular portion of membrane-bound gp130 linked to the Fc region of human IgG1 (sgp130Fc) was developed, and shown to block IL-6 trans-signaling effectively without inhibiting classical IL-6 signaling via membrane-bound gp130 [77] and strongly inhibit colon cancer growth [73]. IL-6 classic signaling has been suggested to be anti-inflammatory, and IL-6 trans-signaling pro-inflammatory by recruiting mononuclear cells and suppressing T cell apoptosis/differentiation [80,81]. IL-6-mediated chemotherapy resistance has been documented in various epithelial cancers [82,83,84]. IL-6 was reported to inhibit radiation-induced apoptosis and promote survival in pancreatic cancer cells by upregulating Bcl-XL [32,85]. Benzyl isothiocyanate-induced decreases in the levels of phosphorylated and total STAT3, a downstream effector of IL-6, were shown to promote apoptosis in pancreatic cancer cells [86]. IL-6 has also been demonstrated to act through the MAPK pathway in pancreatic cancer cells, thereby promoting cell survival [86,87]. It is conceivable that persistently elevated plasma IL-6 levels promote pancreatic cancer cell survival and a pro-inflammatory tumor microenvironment, and mediate resistance to radiotherapy and chemotherapy, thereby negatively impacting survival of PDAC patients. 

### 3.3. Effects of Radiotherapy on Circulating Cytokines

Clinical studies that investigate changes in circulating cytokines following radiotherapy and how such changes may influence tumor response to radiation, local control, and/or treatment-related toxicities in PDAC patients are very limited. In a recent study, Lee et al. [88] demonstrated significantly higher levels of IFN-γ, IL-15, TGF-β, and PDGF-BB in the peritoneal fluids of PDAC patients who received intraoperative radiotherapy (IORT; 10 Gy in 1 fraction) to the surgical bed immediately after resection than those who did not. Interestingly, the peritoneal fluids from IORT-treated patients were shown to suppress proliferation, migration, and invasion of cultured pancreatic cancer cells in vitro by regulating the expression of epithelial-mesenchymal transition markers [88]. Furthermore, cytotoxic and helper T cell populations as well as NK cell population increase at a higher rate while regulatory T cell population maintains at a low ratio in the postoperative period in PDAC patients who received IORT compared to those who did not. These data suggest that IORT causes the release of cytokines and establishes a proinflammatory environment that attracts cytotoxic T cells and NK cells, thereby triggering an antitumor immune response and perhaps contributing to better local control in resected PDAC [88].

SABR, which uses high dose per fraction, achieves secondary tumor cell death by inducing tumor-associated endothelial cell death [89,90] and vascular damage [89,91], in addition to direct tumor cell kill by generating DNA strand breaks. Preclinical data suggest that massive tumor cell death following high dose per fraction SABR leads to the release of tumor antigens and inflammatory cytokines, thereby stimulating an anti-tumor immune response [89,92]. It has been postulated that radiation can modify the immune system within the tumor microenvironment and in the systemic circulation [89,92]. Killing of tumor cells by radiation releases a group of tumor antigens and molecules, collectively known as damage-associated molecular patterns (DAMPS), which in turn, stimulate the expression of immunomodulatory cytokines and produce a pro-inflammatory local and systemic environment [89,92]. Radiation also causes increased extravasation of antigen presenting cells and effector T cells by increasing tumor vascular permeability [92]. Interestingly, multi-fraction SABR has been reported to cause significantly less severe lymphopenia than conventional fractionation radiotherapy with concurrent chemotherapy in patients with unresectable pancreatic cancer [93,94]. Lymphocytes are exquisitely radiosensitive, and radiation-induced lymphopenia is likely the result of direct toxicity to the lymphocytes as they circulate and traverse through the irradiated field. The lymphocyte sparing effect of multi-fraction SABR appeared to be independent of the use of chemotherapy, and could be explained by smaller irradiated tissue and blood volume during SABR [93,95]. The immunomodulatory effects of SABR have sparked interests in combining SABR with immunotherapy to treat various malignancies [96,97,98,99]. The optimal dose fractionation schedule or sequence of treatment for this combinatorial strategy remains to be defined. Emerging data suggest that factors including radiotherapy delivery technique and fractionation strongly influence the ability of SABR to achieve clinically meaningful tumor-specific immune response [100]. 

Our group recently demonstrated that in hepatocellular carcinoma patients undergoing SABR, higher levels of sTNFRII and lower levels of sCD40L and CXCL1 in the circulation after one or two of the planned six fractions correlated with the development of liver toxicity at 3 months post SABR [48]. There was an association between high plasma levels of sTNFRII and sIL-6R early during SABR and increased risk of death at 3 months post treatment [49]. Tumor response at 3 months post SABR was not associated with the circulating levels of the studied cytokines and soluble cytokine receptors early during treatment [49]. These findings suggest that the development of liver toxicity and increased risk of early death following SABR is associated with a pro-inflammatory systemic environment that is mediated in part by cytokines and their soluble receptors. It is worth mentioning that changes in the levels of soluble cytokine receptors were detectable early during the radiation treatment course (i.e., after only one or two of the planned six fractions), potentially allowing radiation dose de-escalation for the remaining fractions or introducing mitigating pharmacological agents to minimize toxicity without compromising local control in the future. It is reasonable to hypothesize that SABR may also elicit a pro-inflammatory milieu and facilitate an antitumor immune response in PDAC patients. 

## 4. Conclusions

Inflammatory cytokines produced by pancreatic cancer cells and stromal cells play an important role in promoting cancer cell survival and mediating an immunosuppressive tumor microenvironment as well as radiotherapy/chemotherapy resistance. High circulating levels of specific cytokines such as IL-6 are associated with a worse prognosis. Antibody drugs have been developed to target cytokines or their receptors. For example, the humanized anti-IL-6R antibody tocilizumab is FDA-approved for the treatment of rheumatoid arthritis, certain autoimmune disease, and severe or life-threatening CAR-T cell-induced cytokine release syndrome [61,101,102]. SABR has the potential to elicit changes in the levels of plasma cytokines or soluble cytokine receptors and stimulate antitumor immune response. Taken together, it would be interesting to explore the combination of SABR and antibody drugs that target cytokines or their receptors such as tocilizumab in the treatment of PDAC. A phase II study, that evaluates the safety and efficacy of tocilizumab, ipilimumab, and nivolumab in combination with SABR (15 Gy in one fraction) in patients with locally advanced or metastatic PDAC, has completed accrual (TRIPPLE-R; NCT04258150); results are pending. The mechanisms of action of SABR in combination with targeted agents and immunotherapy is an important area of research and remain to be fully elucidated. Incorporating correlative studies that examine changes in the circulating cytokinome in prospective ablative radiotherapy clinical trials will help identify the cocktail of cytokines for pharmacological modulation and develop new combinations of treatments involving SABR and targeted agents to improve the clinical outcomes of locally advanced/unresectable or medically inoperable PDAC patients. 

## Data Availability

Not applicable.

## References

[1] Rosenberg L. (1997). Treatment of Pancreatic Cancer. Promises and Problems of Tamoxifen, Somatostatin Analogs, and Gemcitabine. Int. J. Pancreatol..

[2] Siegel R.L., Miller K.D., Fuchs H.E., Jemal A. (2022). Cancer Statistics, 2022. CA Cancer J. Clin..

[3] https://cancer.ca/en/research/cancer-statistics.

[4] Gudjonsson B. (2016). Pancreatic Cancer: 80 Years of Surgery-Percentage and Repetitions. HPB Surg..

[5] Griffin J.F., Smalley S.R., Jewell W., Paradelo J.C., Reymond R.D., Hassanein R.E., Evans R.G. (1990). Patterns of Failure after Curative Resection of Pancreatic Carcinoma. Cancer.

[6] Nitecki S.S., Sarr M.G., Colby T.V., van Heerden J.A. (1995). Long-Term Survival after Resection for Ductal Adenocarcinoma of the Pancreas. Is It Really Improving?. Ann. Surg..

[7] Tepper J., Nardi G., Sutt H. (1976). Carcinoma of the Pancreas: Review of MGH Experience from 1963 to 1973. Analysis of Surgical Failure and Implications for Radiation Therapy. Cancer.

[8] Heinrich S., Pestalozzi B.C., Schäfer M., Weber A., Bauerfeind P., Knuth A., Clavien P.-A. (2008). Prospective Phase II Trial of Neoadjuvant Chemotherapy with Gemcitabine and Cisplatin for Resectable Adenocarcinoma of the Pancreatic Head. J. Clin. Oncol..

[9] Conroy T., Desseigne F., Ychou M., Bouché O., Guimbaud R., Bécouarn Y., Adenis A., Raoul J.-L., Gourgou-Bourgade S., de la Fouchardière C. (2011). FOLFIRINOX versus Gemcitabine for Metastatic Pancreatic Cancer. N. Engl. J. Med..

[10] Von Hoff D.D., Ervin T., Arena F.P., Chiorean E.G., Infante J., Moore M., Seay T., Tjulandin S.A., Ma W.W., Saleh M.N. (2013). Increased Survival in Pancreatic Cancer with Nab-Paclitaxel plus Gemcitabine. N. Engl. J. Med..

[11] https://www.nccn.org/guidelines/category_1.

[12] Iacobuzio-Donahue C.A., Fu B., Yachida S., Luo M., Abe H., Henderson C.M., Vilardell F., Wang Z., Keller J.W., Banerjee P. (2009). DPC4 Gene Status of the Primary Carcinoma Correlates with Patterns of Failure in Patients with Pancreatic Cancer. J. Clin. Oncol..

[13] Moertel C.G., Frytak S., Hahn R.G., O’Connell M.J., Reitemeier R.J., Rubin J., Schutt A.J., Weiland L.H., Childs D.S., Holbrook M.A. (1981). Therapy of Locally Unresectable Pancreatic Carcinoma: A Randomized Comparison of High Dose (6000 Rads) Radiation Alone, Moderate Dose Radiation (4000 Rads + 5-Fluorouracil), and High Dose Radiation + 5-Fluorouracil: The Gastrointestinal Tumor Study Group. Cancer.

[14] Chauffert B., Mornex F., Bonnetain F., Rougier P., Mariette C., Bouché O., Bosset J.F., Aparicio T., Mineur L., Azzedine A. (2008). Phase III Trial Comparing Intensive Induction Chemoradiotherapy (60 Gy, Infusional 5-FU and Intermittent Cisplatin) Followed by Maintenance Gemcitabine with Gemcitabine Alone for Locally Advanced Unresectable Pancreatic Cancer. Definitive Results of the 2000-01 FFCD/SFRO Study. Ann. Oncol..

[15] Loehrer P.J., Feng Y., Cardenes H., Wagner L., Brell J.M., Cella D., Flynn P., Ramanathan R.K., Crane C.H., Alberts S.R. (2011). Gemcitabine Alone versus Gemcitabine plus Radiotherapy in Patients with Locally Advanced Pancreatic Cancer: An Eastern Cooperative Oncology Group Trial. J. Clin. Oncol..

[16] Krishnan S., Rana V., Janjan N.A., Varadhachary G.R., Abbruzzese J.L., Das P., Delclos M.E., Gould M.S., Evans D.B., Wolff R.A. (2007). Induction Chemotherapy Selects Patients with Locally Advanced, Unresectable Pancreatic Cancer for Optimal Benefit from Consolidative Chemoradiation Therapy. Cancer.

[17] Hammel P., Huguet F., van Laethem J.-L., Goldstein D., Glimelius B., Artru P., Borbath I., Bouché O., Shannon J., André T. (2016). Effect of Chemoradiotherapy vs Chemotherapy on Survival in Patients With Locally Advanced Pancreatic Cancer Controlled After 4 Months of Gemcitabine With or Without Erlotinib: The LAP07 Randomized Clinical Trial. JAMA.

[18] Koay E.J., Hanania A.N., Hall W.A., Taniguchi C.M., Rebueno N., Myrehaug S., Aitken K.L., Dawson L.A., Crane C.H., Herman J.M. (2020). Dose-Escalated Radiation Therapy for Pancreatic Cancer: A Simultaneous Integrated Boost Approach. Pract. Radiat. Oncol..

[19] Rosati L.M., Kumar R., Herman J.M. (2017). Integration of Stereotactic Body Radiation Therapy into the Multidisciplinary Management of Pancreatic Cancer. Semin. Radiat. Oncol..

[20] Moningi S., Raman S.P., Dholakia A.S., Hacker-Prietz A., Pawlik T.M., Zheng L., Weiss M., Laheru D.A., Wolfgang C.L., Herman J.M. (2014). Stereotactic Body Radiation Therapy for Pancreatic Cancer: Single Institutional Experience. J. Clin. Oncol..

[21] Pollom E.L., Alagappan M., Chan C., Shultz D., Kunz P.L., Koong A., Chang D.T. (2014). Outcomes and Toxicity of SBRT for Patients with Unresectable Pancreatic Adenocarcinoma. J. Clin. Oncol..

[22] Herman J.M., Chang D.T., Goodman K.A., Dholakia A.S., Raman S.P., Hacker-Prietz A., Iacobuzio-Donahue C.A., Griffith M.E., Pawlik T.M., Pai J.S. (2015). Phase 2 Multi-Institutional Trial Evaluating Gemcitabine and Stereotactic Body Radiotherapy for Patients with Locally Advanced Unresectable Pancreatic Adenocarcinoma. Cancer.

[23] Mahadevan A., Miksad R., Goldstein M., Sullivan R., Bullock A., Buchbinder E., Pleskow D., Sawhney M., Kent T., Vollmer C. (2011). Induction Gemcitabine and Stereotactic Body Radiotherapy for Locally Advanced Nonmetastatic Pancreas Cancer. Int. J. Radiat. Oncol. Biol. Phys..

[24] Petrelli F., Comito T., Ghidini A., Torri V., Scorsetti M., Barni S. (2017). Stereotactic Body Radiation Therapy for Locally Advanced Pancreatic Cancer: A Systematic Review and Pooled Analysis of 19 Trials. Int. J. Radiat. Oncol. Biol. Phys..

[25] Moningi S., Dholakia A.S., Raman S.P., Blackford A., Cameron J.L., Le D.T., De Jesus-Acosta A.M.C., Hacker-Prietz A., Rosati L.M., Assadi R.K. (2015). The Role of Stereotactic Body Radiation Therapy for Pancreatic Cancer: A Single-Institution Experience. Ann. Surg. Oncol..

[26] Palta M., Godfrey D., Goodman K.A., Hoffe S., Dawson L.A., Dessert D., Hall W.A., Herman J.M., Khorana A.A., Merchant N. (2019). Radiation Therapy for Pancreatic Cancer: Executive Summary of an ASTRO Clinical Practice Guideline. Pract. Radiat. Oncol..

[27] Caravatta L., Cellini F., Simoni N., Rosa C., Niespolo R.M., Lupattelli M., Picardi V., Macchia G., Sainato A., Mantello G. (2019). Magnetic Resonance Imaging (MRI) Compared with Computed Tomography (CT) for Interobserver Agreement of Gross Tumor Volume Delineation in Pancreatic Cancer: A Multi-Institutional Contouring Study on Behalf of the AIRO Group for Gastrointestinal Cancers. Acta Oncol..

[28] Chuong M.D., Bryant J., Mittauer K.E., Hall M., Kotecha R., Alvarez D., Romaguera T., Rubens M., Adamson S., Godley A. (2021). Ablative 5-Fraction Stereotactic Magnetic Resonance-Guided Radiation Therapy With On-Table Adaptive Replanning and Elective Nodal Irradiation for Inoperable Pancreas Cancer. Pract. Radiat. Oncol..

[29] Chuong M.D., Herrera R., Kaiser A., Rubens M., Romaguera T., Alvarez D., Kotecha R., Hall M.D., McCulloch J., Ucar A. (2022). Induction Chemotherapy and Ablative Stereotactic Magnetic Resonance Image-Guided Adaptive Radiation Therapy for Inoperable Pancreas Cancer. Front. Oncol..

[30] Reyngold M., O’Reilly E.M., Varghese A.M., Fiasconaro M., Zinovoy M., Romesser P.B., Wu A., Hajj C., Cuaron J.J., Tuli R. (2021). Association of Ablative Radiation Therapy With Survival Among Patients With Inoperable Pancreatic Cancer. JAMA Oncol..

[31] Hammer L., Hausner D., Ben-Ayun M., Shacham-Shmueli E., Morag O., Margalit O., Boursi B., Yarom N., Jacobson G., Katzman T. (2022). Single-Fraction Celiac Plexus Radiosurgery: A Preliminary Proof-of-Concept Phase 2 Clinical Trial. Int. J. Radiat. Oncol. Biol. Phys..

[32] Ebrahimi B., Tucker S.L., Li D., Abbruzzese J.L., Kurzrock R. (2004). Cytokines in Pancreatic Carcinoma: Correlation with Phenotypic Characteristics and Prognosis. Cancer.

[33] Babic A., Schnure N., Neupane N.P., Zaman M.M., Rifai N., Welch M.W., Brais L.K., Rubinson D.A., Morales-Oyarvide V., Yuan C. (2018). Plasma Inflammatory Cytokines and Survival of Pancreatic Cancer Patients. Clin. Transl. Gastroenterol..

[34] Bellone G., Smirne C., Mauri F.A., Tonel E., Carbone A., Buffolino A., Dughera L., Robecchi A., Pirisi M., Emanuelli G. (2006). Cytokine Expression Profile in Human Pancreatic Carcinoma Cells and in Surgical Specimens: Implications for Survival. Cancer Immunol. Immunother..

[35] Falconer J.S., Fearon K.C., Plester C.E., Ross J.A., Carter D.C. (1994). Cytokines, the Acute-Phase Response, and Resting Energy Expenditure in Cachectic Patients with Pancreatic Cancer. Ann. Surg..

[36] Okada S., Okusaka T., Ishii H., Kyogoku A., Yoshimori M., Kajimura N., Yamaguchi K., Kakizoe T. (1998). Elevated Serum Interleukin-6 Levels in Patients with Pancreatic Cancer. Jpn. J. Clin. Oncol..

[37] Dima S.O., Tanase C., Albulescu R., Herlea V., Chivu-Economescu M., Purnichescu-Purtan R., Dumitrascu T., Duda D.G., Popescu I. (2012). An Exploratory Study of Inflammatory Cytokines as Prognostic Biomarkers in Patients with Ductal Pancreatic Adenocarcinoma. Pancreas.

[38] Lippitz B.E. (2013). Cytokine Patterns in Patients with Cancer: A Systematic Review. Lancet Oncol..

[39] Roshani R., McCarthy F., Hagemann T. (2014). Inflammatory Cytokines in Human Pancreatic Cancer. Cancer Lett..

[40] Bissell M.J., Radisky D. (2001). Putting Tumours in Context. Nat. Rev. Cancer.

[41] Mueller M.M., Fusenig N.E. (2004). Friends or Foes—Bipolar Effects of the Tumour Stroma in Cancer. Nat. Rev. Cancer.

[42] Micke P., Ostman A. (2004). Tumour-Stroma Interaction: Cancer-Associated Fibroblasts as Novel Targets in Anti-Cancer Therapy?. Lung Cancer.

[43] Hojilla C.V., Mohammed F.F., Khokha R. (2003). Matrix Metalloproteinases and Their Tissue Inhibitors Direct Cell Fate during Cancer Development. Br. J. Cancer.

[44] Vacchelli E., Galluzzi L., Eggermont A., Galon J., Tartour E., Zitvogel L., Kroemer G. (2012). Trial Watch: Immunostimulatory Cytokines. Oncoimmunology.

[45] Dendorfer U. (1996). Molecular Biology of Cytokines. Artif. Organs.

[46] Lierova A., Jelicova M., Nemcova M., Proksova M., Pejchal J., Zarybnicka L., Sinkorova Z. (2018). Cytokines and Radiation-Induced Pulmonary Injuries. J. Radiat. Res..

[47] Torres C., Linares A., Alejandre M.J., Palomino-Morales R.J., Caba O., Prados J., Aránega A., Delgado J.R., Irigoyen A., Martínez-Galán J. (2015). Prognosis Relevance of Serum Cytokines in Pancreatic Cancer. Biomed Res. Int..

[48] van der Sijde F., Mustafa D.A.M., Vietsch E.E., Katsikis P.D., van Eijck C.H.J. (2021). Circulating Immunological Biomarkers: Prognosis of Pancreatic Cancer Patients Reflected by the Immune System. Pancreas.

[49] Ng S.S.W., Zhang H., Wang L., Citrin D., Dawson L.A. (2020). Association of pro-Inflammatory Soluble Cytokine Receptors Early during Hepatocellular Carcinoma Stereotactic Radiotherapy with Liver Toxicity. NPJ Precis. Oncol..

[50] Abbate A., Toldo S., Marchetti C., Kron J., Van Tassell B.W., Dinarello C.A. (2020). Interleukin-1 and the Inflammasome as Therapeutic Targets in Cardiovascular Disease. Circ. Res..

[51] Park W., Chawla A., O’Reilly E.M. (2021). Pancreatic Cancer: A Review. JAMA.

[52] Bardeesy N., DePinho R.A. (2002). Pancreatic Cancer Biology and Genetics. Nat. Rev. Cancer.

[53] Protti M.P., De Monte L. (2013). Immune Infiltrates as Predictive Markers of Survival in Pancreatic Cancer Patients. Front. Physiol..

[54] Neesse A., Bauer C.A., Öhlund D., Lauth M., Buchholz M., Michl P., Tuveson D.A., Gress T.M. (2019). Stromal Biology and Therapy in Pancreatic Cancer: Ready for Clinical Translation?. Gut.

[55] Neesse A., Algül H., Tuveson D.A., Gress T.M. (2015). Stromal Biology and Therapy in Pancreatic Cancer: A Changing Paradigm. Gut.

[56] Chu G.C., Kimmelman A.C., Hezel A.F., DePinho R.A. (2007). Stromal Biology of Pancreatic Cancer. J. Cell. Biochem..

[57] Mahadevan D., Von Hoff D.D. (2007). Tumor-Stroma Interactions in Pancreatic Ductal Adenocarcinoma. Mol. Cancer Ther..

[58] Ren C., Chen Y., Han C., Fu D., Chen H. (2014). Plasma Interleukin-11 (IL-11) Levels Have Diagnostic and Prognostic Roles in Patients with Pancreatic Cancer. Tumour Biol..

[59] Mitsunaga S., Ikeda M., Shimizu S., Ohno I., Furuse J., Inagaki M., Higashi S., Kato H., Terao K., Ochiai A. (2013). Serum Levels of IL-6 and IL-1β Can Predict the Efficacy of Gemcitabine in Patients with Advanced Pancreatic Cancer. Br. J. Cancer.

[60] van der Sijde F., Dik W.A., Mustafa D.A.M., Vietsch E.E., Besselink M.G., Debets R., Koerkamp B.G., Haberkorn B.C.M., Homs M.Y.V., Janssen Q.P. (2022). Serum Cytokine Levels Are Associated with Tumor Progression during FOLFIRINOX Chemotherapy and Overall Survival in Pancreatic Cancer Patients. Front. Immunol..

[61] Nishimoto N., Kishimoto T. (2006). Interleukin 6: From Bench to Bedside. Nat. Clin. Pract. Rheumatol..

[62] Taga T. (1992). IL6 Signalling through IL6 Receptor and Receptor-Associated Signal Transducer, gp130. Res. Immunol..

[63] Chalaris A., Garbers C., Rabe B., Rose-John S., Scheller J. (2011). The Soluble Interleukin 6 Receptor: Generation and Role in Inflammation and Cancer. Eur. J. Cell Biol..

[64] Rose-John S., Waetzig G.H., Scheller J., Grötzinger J., Seegert D. (2007). The IL-6/sIL-6R Complex as a Novel Target for Therapeutic Approaches. Expert Opin. Ther. Targets.

[65] Nakahara H., Song J., Sugimoto M., Hagihara K., Kishimoto T., Yoshizaki K., Nishimoto N. (2003). Anti-Interleukin-6 Receptor Antibody Therapy Reduces Vascular Endothelial Growth Factor Production in Rheumatoid Arthritis. Arthritis Rheum..

[66] Puthier D., Derenne S., Barillé S., Moreau P., Harousseau J.L., Bataille R., Amiot M. (1999). Mcl-1 and Bcl-xL Are Co-Regulated by IL-6 in Human Myeloma Cells. Br. J. Haematol..

[67] Spets H., Strömberg T., Georgii-Hemming P., Siljason J., Nilsson K., Jernberg-Wiklund H. (2002). Expression of the Bcl-2 Family of pro- and Anti-Apoptotic Genes in Multiple Myeloma and Normal Plasma Cells: Regulation during Interleukin-6(IL-6)-Induced Growth and Survival. Eur. J. Haematol..

[68] Hodge D.R., Hurt E.M., Farrar W.L. (2005). The Role of IL-6 and STAT3 in Inflammation and Cancer. Eur. J. Cancer.

[69] Yang L., Wang L., Lin H.-K., Kan P.-Y., Xie S., Tsai M.-Y., Wang P.-H., Chen Y.-T., Chang C. (2003). Interleukin-6 Differentially Regulates Androgen Receptor Transactivation via PI3K-Akt, STAT3, and MAPK, Three Distinct Signal Pathways in Prostate Cancer Cells. Biochem. Biophys. Res. Commun..

[70] Nakanishi H., Yoshioka K., Joyama S., Araki N., Myoui A., Ishiguro S., Ueda T., Yoshikawa H., Itoh K. (2004). Interleukin-6/soluble Interleukin-6 Receptor Signaling Attenuates Proliferation and Invasion, and Induces Morphological Changes of a Newly Established Pleomorphic Malignant Fibrous Histiocytoma Cell Line. Am. J. Pathol..

[71] Müllberg J., Dittrich E., Graeve L., Gerhartz C., Yasukawa K., Taga T., Kishimoto T., Heinrich P.C., Rose-John S. (1993). Differential Shedding of the Two Subunits of the Interleukin-6 Receptor. FEBS Lett..

[72] Müllberg J., Schooltink H., Stoyan T., Günther M., Graeve L., Buse G., Mackiewicz A., Heinrich P.C., Rose-John S. (1993). The Soluble Interleukin-6 Receptor Is Generated by Shedding. Eur. J. Immunol..

[73] Matthews V., Schuster B., Schütze S., Bussmeyer I., Ludwig A., Hundhausen C., Sadowski T., Saftig P., Hartmann D., Kallen K.-J. (2003). Cellular Cholesterol Depletion Triggers Shedding of the Human Interleukin-6 Receptor by ADAM10 and ADAM17 (TACE). J. Biol. Chem..

[74] Horiuchi S., Koyanagi Y., Zhou Y., Miyamoto H., Tanaka Y., Waki M., Matsumoto A., Yamamoto M., Yamamoto N. (1994). Soluble Interleukin-6 Receptors Released from T Cell or Granulocyte/macrophage Cell Lines and Human Peripheral Blood Mononuclear Cells Are Generated through an Alternative Splicing Mechanism. Eur. J. Immunol..

[75] Scheller J., Ohnesorge N., Rose-John S. (2006). Interleukin-6 Trans-Signalling in Chronic Inflammation and Cancer. Scand. J. Immunol..

[76] Peters M., Müller A.M., Rose-John S. (1998). Interleukin-6 and Soluble Interleukin-6 Receptor: Direct Stimulation of gp130 and Hematopoiesis. Blood.

[77] Jostock T., Müllberg J., Ozbek S., Atreya R., Blinn G., Voltz N., Fischer M., Neurath M.F., Rose-John S. (2001). Soluble gp130 Is the Natural Inhibitor of Soluble Interleukin-6 Receptor Transsignaling Responses. Eur. J. Biochem..

[78] Narazaki M., Yasukawa K., Saito T., Ohsugi Y., Fukui H., Koishihara Y., Yancopoulos G.D., Taga T., Kishimoto T. (1993). Soluble Forms of the Interleukin-6 Signal-Transducing Receptor Component gp130 in Human Serum Possessing a Potential to Inhibit Signals through Membrane-Anchored gp130. Blood.

[79] Becker C., Fantini M.C., Wirtz S., Nikolaev A., Lehr H.A., Galle P.R., Rose-John S., Neurath M.F. (2005). IL-6 Signaling Promotes Tumor Growth in Colorectal Cancer. Cell Cycle.

[80] Scheller J., Chalaris A., Schmidt-Arras D., Rose-John S. (2011). The pro- and Anti-Inflammatory Properties of the Cytokine Interleukin-6. Biochim. Biophys. Acta.

[81] Rose-John S. (2017). The Soluble Interleukin 6 Receptor: Advanced Therapeutic Options in Inflammation. Clin. Pharmacol. Ther..

[82] Domingo-Domenech J., Oliva C., Rovira A., Codony-Servat J., Bosch M., Filella X., Montagut C., Tapia M., Campás C., Dang L. (2006). Interleukin 6, a Nuclear Factor-kappaB Target, Predicts Resistance to Docetaxel in Hormone-Independent Prostate Cancer and Nuclear Factor-kappaB Inhibition by PS-1145 Enhances Docetaxel Antitumor Activity. Clin. Cancer Res..

[83] Duan Z., Foster R., Bell D.A., Mahoney J., Wolak K., Vaidya A., Hampel C., Lee H., Seiden M.V. (2006). Signal Transducers and Activators of Transcription 3 Pathway Activation in Drug-Resistant Ovarian Cancer. Clin. Cancer Res..

[84] Ikuta K., Takemura K., Kihara M., Nishimura M., Ueda N., Naito S., Lee E., Shimizu E., Yamauchi A. (2005). Overexpression of Constitutive Signal Transducer and Activator of Transcription 3 mRNA in Cisplatin-Resistant Human Non-Small Cell Lung Cancer Cells. Oncol. Rep..

[85] Miyamoto Y., Hosotani R., Doi R., Wada M., Ida J., Tsuji S., Kawaguchi M., Nakajima S., Kobayashi H., Masui T. (2001). Interleukin-6 Inhibits Radiation Induced Apoptosis in Pancreatic Cancer Cells. Anticancer Res..

[86] Sahu R.P., Srivastava S.K. (2009). The Role of STAT-3 in the Induction of Apoptosis in Pancreatic Cancer Cells by Benzyl Isothiocyanate. J. Natl. Cancer Inst..

[87] Feurino L.W., Zhang Y., Bharadwaj U., Zhang R., Li F., Fisher W.E., Brunicardi F.C., Chen C., Yao Q., Min L. (2007). IL-6 Stimulates Th2 Type Cytokine Secretion and Upregulates VEGF and NRP-1 Expression in Pancreatic Cancer Cells. Cancer Biol. Ther..

[88] Lee Y.S., Kim H.S., Cho Y., Lee I.J., Kim H.J., Lee D.E., Kang H.W., Park J.S. (2021). Intraoperative Radiation Therapy Induces Immune Response Activity after Pancreatic Surgery. BMC Cancer.

[89] Song C.W., Kim M.-S., Cho L.C., Dusenbery K., Sperduto P.W. (2014). Radiobiological Basis of SBRT and SRS. Int. J. Clin. Oncol..

[90] Garcia-Barros M. (2003). Tumor Response to Radiotherapy Regulated by Endothelial Cell Apoptosis. Science.

[91] Park H.J., Griffin R.J., Hui S., Levitt S.H., Song C.W. (2012). Radiation-Induced Vascular Damage in Tumors: Implications of Vascular Damage in Ablative Hypofractionated Radiotherapy (SBRT and SRS). Radiat. Res..

[92] Finkelstein S.E., Timmerman R., McBride W.H., Schaue D., Hoffe S.E., Mantz C.A., Wilson G.D. (2011). The Confluence of Stereotactic Ablative Radiotherapy and Tumor Immunology. Clin. Dev. Immunol..

[93] Wild A.T., Herman J.M., Dholakia A.S., Moningi S., Lu Y., Rosati L.M., Hacker-Prietz A., Assadi R.K., Saeed A.M., Pawlik T.M. (2016). Lymphocyte-Sparing Effect of Stereotactic Body Radiation Therapy in Patients With Unresectable Pancreatic Cancer. Int. J. Radiat. Oncol. Biol. Phys..

[94] Wu G., Baine M.J., Zhao N., Li S., Li X., Lin C. (2019). Lymphocyte-Sparing Effect of Stereotactic Body Radiation Therapy Compared to Conventional Fractionated Radiation Therapy in Patients with Locally Advanced Pancreatic Cancer. BMC Cancer.

[95] Ellsworth S.G. (2018). Field Size Effects on the Risk and Severity of Treatment-Induced Lymphopenia in Patients Undergoing Radiation Therapy for Solid Tumors. Adv. Radiat. Oncol..

[96] Formenti S.C., Demaria S. (2013). Combining Radiotherapy and Cancer Immunotherapy: A Paradigm Shift. J. Natl. Cancer Inst..

[97] Grassberger C., Ellsworth S.G., Wilks M.Q., Keane F.K., Loeffler J.S. (2019). Assessing the Interactions between Radiotherapy and Antitumour Immunity. Nat. Rev. Clin. Oncol..

[98] Luke J.J., Lemons J.M., Karrison T.G., Pitroda S.P., Melotek J.M., Zha Y., Al-Hallaq H.A., Arina A., Khodarev N.N., Janisch L. (2018). Safety and Clinical Activity of Pembrolizumab and Multisite Stereotactic Body Radiotherapy in Patients With Advanced Solid Tumors. J. Clin. Oncol..

[99] Bahig H., Aubin F., Stagg J., Gologan O., Ballivy O., Bissada E., Nguyen-Tan F.-P., Soulières D., Guertin L., Filion E. (2019). Phase I/II Trial of Durvalumab plus Tremelimumab and Stereotactic Body Radiotherapy for Metastatic Head and Neck Carcinoma. BMC Cancer.

[100] Tubin S., Yan W., Mourad W.F., Fossati P., Khan M.K. (2020). The Future of Radiation-Induced Abscopal Response: Beyond Conventional Radiotherapy Approaches. Future Oncol..

[101] Mima T., Nishimoto N. (2009). Clinical Value of Blocking IL-6 Receptor. Curr. Opin. Rheumatol..

[102] Le R.Q., Li L., Yuan W., Shord S.S., Nie L., Habtemariam B.A., Przepiorka D., Farrell A.T., Pazdur R. (2018). FDA Approval Summary: Tocilizumab for Treatment of Chimeric Antigen Receptor T Cell-Induced Severe or Life-Threatening Cytokine Release Syndrome. Oncologist.

